# Host-Dependent Phytochemical Profiles and Antioxidant and Hypoglycemic Activities of Mexican Mistletoe (*Psittacanthus calyculatus*) Organs

**DOI:** 10.3390/molecules30214257

**Published:** 2025-10-31

**Authors:** Zaida Ochoa-Cruz, Jorge Molina-Torres, Hortencia Gabriela Mena-Violante, Jeanette Guadalupe Cárdenas-Valdovinos, Mariana Villa-Santiago, María Valentina Angoa-Pérez

**Affiliations:** 1 Instituto Politécnico Nacional, Centro Interdisciplinario de Investigación para el Desarrollo Integral Regional Unidad Michoacan (CIIIDIR-IPN), Department of Research, Jiquilapan 59510, Mexico; zochoac1900@alumno.ipn.mx (Z.O.-C.); hmena@ipn.mx (H.G.M.-V.); jcardenasv@ipn.mx (J.G.C.-V.); mvillas1900@alumno.ipn.mx (M.V.-S.); 2 Instituto Politécnico Nacional, Centro de Investigación de Estudios Avanzados Unidad Irapuato (CINVESTAV-IPN), Department of Biotechnology and Biochemistry, Irapuato 36821, Mexico; jmolina@cinvestav.mx

**Keywords:** Mexican mistletoe, parasite–host interaction, freeze-dried organs, phytochemistry, antioxidant activity, α-glucosidase inhibition

## Abstract

Mexican mistletoe (*Psittacanthus calyculatus*) is an ecologically and pharmacologically relevant hemiparasitic plant whose phytochemical composition varies according to host, organ, and processing. This study analyzed the pericarp, flower, leaf, and peduncle in fresh and freeze-dried conditions. The samples were collected from *Forestiera phillyreoides* and *Mimosa* sp. High-performance thin-layer chromatography revealed the presence of malvidin-3-O-glucoside (27.43 ± 1.88 mg/g dry weight [DW]) in freeze-dried pericarps, cyanidin-3-O-glucoside (26.55 ± 1.19 mg/g DW) in freeze-dried flowers, and rutin (5.39 ± 1.24 mg/g DW) in freeze-dried leaves collected from *Mimosa* sp. Gas chromatography coupled with mass spectrometry (GC-MS) confirmed the presence of gallic acid (40.40 ± 0.228 mg/g DW in freeze-dried pericarps of *Mimosa* sp.), which was 42.9% higher than the amount found in *F. phillyreoides* plants. Regarding antioxidant activity, freeze-dried mistletoe pericarps collected from *Mimosa* sp. exhibited the highest capacity (85.7–94.9% DPPH· and ABTS·^+^ inhibition, respectively). For α-glucosidase inhibition, the half-maximal inhibitory concentration (IC_50_) values of freeze-dried flowers and pericarps were low (84–85 μg/mL), comparable to acarbose (62 μg/mL). Freeze-drying increased metabolite concentration by up to 54% for gallic acid in the pericarp of plants collected from *Mimosa* sp. and enhanced bioactivity. Overall, Mexican mistletoe is established as a nutraceutical source with therapeutic potential and sustainable use value.

## 1. Introduction

Mistletoe is recognized as a diverse group of parasitic or hemiparasitic plants belonging to the families Loranthaceae and Viscaceae. These plants form haustoria to obtain water and nutrients from the host, while many species retain photosynthetic capacity [1,2,3]. *Psittacanthus calyculatus* (DC.) G. Don, which is commonly referred to as the “Mexican mistletoe,” is characterized as a hemiparasitic plant that is distributed from Mexico to northern Argentina. It parasitizes a wide range of species, including oaks (*Quercus* spp.), pines (*Pinus* spp.), mesquites (*Prosopis* spp.), huizaches (*Acacia* spp.), *Mimosa* sp., peach trees (*Prunus* spp.), and avocados (*Persea americana*), among others [4,5,6]. The economic relevance of the species is attributed to the reduction in vigor and productivity of forest, agricultural, and ornamental species. Its ecological importance is associated with its role as a food resource for frugivorous birds and other organisms [5,7].

Notwithstanding the deleterious effects of this species, it has been extensively utilized in Latin American traditional medicine for the treatment of numerous chronic diseases due to the therapeutic effects of its secondary metabolites, including flavonoids, phenolic acids, tannins, and alkaloids [8,9,10]. In studies conducted on other mistletoe species, such as *Phragmanthera incana* and *Viscum album*, it has been documented that their phytochemical and mineral composition exhibits variability depending on the host, thereby influencing their biological activity and therapeutic potential [11,12]. This pattern indicates that the chemical variability of *P. calyculatus* may also be contingent on its host.

Despite the extensive research conducted on the genus *Psittacanthus*, with a predominant focus on foliage as evidenced in the extant literature [13,14], there remains a paucity of attention directed towards the study of fruits. This is particularly salient given their fundamental role in the reproductive cycle and their potential as a source of bioactive compounds, including anthocyanins such as cyanidin-3-glucoside [15]. A comprehensive understanding of its chemical composition across different organs can offer valuable insights for the sustainable management of this species and its hosts. Such knowledge can help avoid complete eradication and instead support the potential use of this species.

In fact, there is a growing body of research highlighting the bioactive properties of specific phenolic compounds in mistletoe species. Anthocyanins, including pelargonidin-3-O-glucoside (Pg-3g), cyanidin-3-O-glucoside (Cy-3g), peonidin-3-O-glucoside (Pe-3g), and malvidin-3-O-glucoside (Mv-3g), are pigments with strong antioxidant capacity and potential relevance in metabolic disorders [10,15,16]. Rutin, a flavonol glycoside with a wide distribution in plants, has been associated with antioxidant, anti-inflammatory, and anti-hyperglycemic activities [17]. Gallic acid, a prominent phenolic acid found in *P. calyculatus*, has been identified for its radical scavenging properties and its role in regulating glucose metabolism [8,15]. These compounds have been identified as potential target analytes because of their representation of significant classes of phenolics, anthocyanins, flavonoids, and phenolic acids. These compounds have been previously associated with the antioxidant and hypoglycemic properties of parasitic plants, thereby serving as reliable markers to assess organ- and host-dependent variability in *P. calyculatus*.

A plethora of studies have previously documented the biological activities of *P. calyculatus* extracts. For instance, methanolic leaf extracts have demonstrated strong antioxidant and anti-genotoxic effects associated with high levels of polyphenols and flavonoids [18]. In streptozotocin-induced diabetic rats, extracts demonstrated a substantial reduction in glycemia without inducing genotoxic effects, thereby substantiating their hypoglycemic potential attributed to tannins, catechins, and gallic acid [8]. A body of literature on mistletoe species related to the subject has indicated the presence of anti-inflammatory and immunomodulatory activities, which have been linked to the presence of triterpenes and flavonoids [19,20,21]. The presented evidence underscores the pharmacological relevance of *P. calyculatus* and emphasizes the necessity of investigating its organ-specific chemical variability and biological properties.

In this context, the present study analyzed the phytochemical profiles of the leaves, flowers, peduncle, and fruits of *P. calyculatus*, as well as their association with antioxidant and hypoglycemic biological activities. This analysis took into consideration the influence of the hosts *Forestiera phillyreoides* and *Mimosa* sp. on which the species develops.

## 2. Results

### 2.1. Anthocyanins and the Flavonoid Detected and Quantified by HPTLC

#### 2.1.1. Anthocyanins Detected in the Pericarp and Flower of *P. calyculatus*

**Figure 1:**
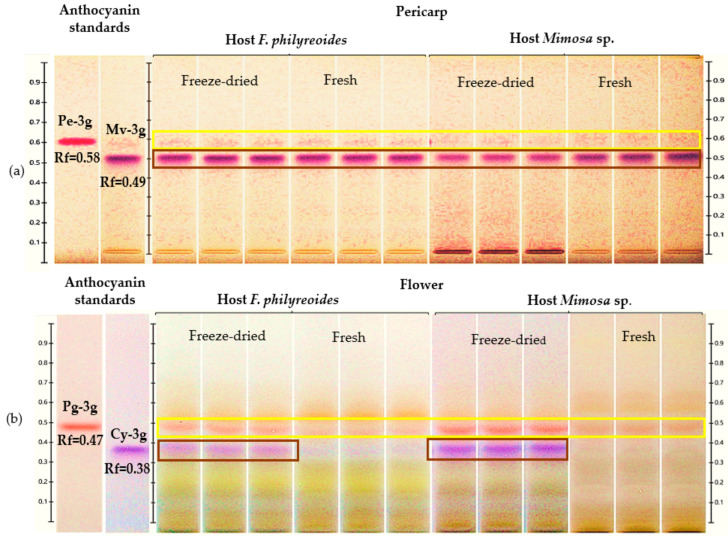
HPTLC chromatogram for the detection and quantification of anthocyanins in freeze-dried and fresh pericarp and flower organs of *P. calyculatus* grown on *F. phillyreoides* and *Mimosa* sp., obtained under white light after derivatization with the NP reagent. Rf, retention factor. (**a**) Detection of peonidin 3-glucoside (Pe-3g, Rf = 0.58) and malvidin 3-glucoside (Mv-3g, Rf = 0.49); (**b**) detection of pelargonidin 3-glucoside (Pg-3g, Rf = 0.47), and cyanidin 3-glucoside (Cy-3g, Rf = 0.38).

The presence of Pe-3g (Rf = 0.58) and Mv-3g (Rf = 0.49) in the pericarp, as well as Pg-3g (Rf = 0.47) and Cy-3g (Rf = 0.38) in the flower, was detected in acidified methanolic extracts of *P. calyculatus* collected from plants parasitizing *F. phillyreoides* and *Mimosa* sp. (Figure 1a and Figure 1b, respectively).

Pe-3g (Rf = 0.58) and Mv-3g (Rf = 0.55) were detected in freeze-dried and fresh pericarps from both hosts (Figure 1a). The presence of Pe-3g was observed at low intensity in both processing states. Conversely, the bands corresponding to Mv-3g exhibited higher intensity in both hosts. This observation suggests that the concentration of this anthocyanin may be elevated or that its stability may be enhanced during the freeze-drying process.

In the floral organ, Pg-3g (Rf = 0.47) and Cy-3g (Rf = 0.38) were detected under both processing conditions (Figure 1b). The freeze-dried flowers of both hosts exhibited stronger and more visible bands, with Cy-3g displaying more intense coloration in *Mimosa* sp. compared to *F. phillyreoides*. The absence of supplementary signals in disparate positions served to substantiate the selectivity of the method for the anthocyanins evaluated. The findings suggest that both the host and the processing state of the organ exerted an influence on anthocyanin concentrations. Furthermore, it was observed that freeze-drying enhanced the detection and quantification of these compounds in *P. calyculatus*.

#### 2.1.2. Flavonoid Detected on the Leaf of *P. calyculatus*

**Figure 2:**
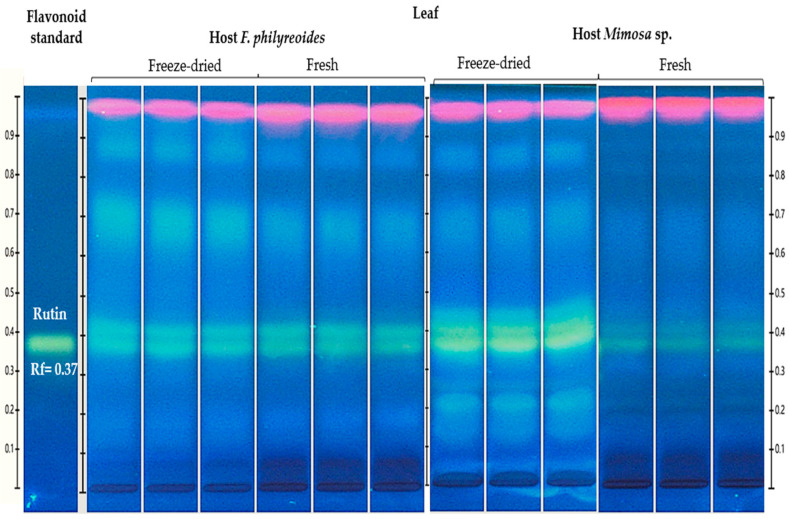
HPTLC chromatogram for the detection and quantification of rutin (Rt, Rf = 0.37) in freeze-dried and fresh organs of *P. calyculatus* grown on *F. phillyreoides* and *Mimosa* sp., obtained under UV light (366 nm) after derivatization with the NP reagent. Rf, retention factor.

The presence of rutin (Rt, Rf = 0.38) was detected in freeze-dried and fresh leaf organs of *P. calyculatus* grown on *F. phillyreoides* and *Mimosa* sp. (Figure 2).

#### 2.1.3. Quantification of Metabolites Detected in *P. calyculatus* by HPTLC

The quantification of metabolites was assessed in the organs of *P. calyculatus* collected from both hosts (*F. phillyreoides* and *Mimosa* sp.), considering two processing states (freeze-dried and fresh organs). These results are presented in Table 1. In the freeze-dried pericarp, the quantification was performed only for Mv-3g, while Pe-3g was detected but did not exceed the quantification threshold. In the floral organ, the quantity of Pg-3g was measured in both freeze-dried and fresh states, while Cy-3g was only measured in freeze-dried organs. In fresh samples, Cy-3g was below the detection limit.

A general tendency was observed indicating higher concentrations in freeze-dried organs in comparison to fresh organs. The highest concentrations of Mv-3g were observed in freeze-dried pericarp from *Mimosa* sp. (27.433 ± 1.887 mg/g DW), followed by Cy-3g in freeze-dried flowers of the same host (26.557 ± 1.192 mg/g DW). In *F. phillyreoides*, the highest concentrations were observed in Cy-3g in freeze-dried flowers (18.597 ± 0.829 mg/g dry weight [DW]) and Mv-3g in fresh pericarp (16.29 ± 0.234 mg/g fresh weight [FW]), although these were lower than those observed in *Mimosa* sp.

In comparison, the freeze-dried pericarp of *Mimosa* sp. exhibited a 54% increase in the level of phenolic compounds compared to that of *F. phillyreoides*, while the freeze-dried flowers of *Mimosa* sp. demonstrated a 43% higher content of phenolics than those of *F. phillyreoides*. In leaves, the highest concentration was observed in freeze-dried organs of *Mimosa* sp. (5.387 ± 1.248 mg/g DW), which is 9.9% higher than in *F. phillyreoides*.

### 2.2. Identification of Compounds by GC–MS

In the absence of a clear separation for the peduncle under HPTLC, the analysis of metabolites was conducted by gas chromatography coupled with mass spectrometry (GC–MS). Given the low volatility and thermal susceptibility of phenolic compounds, derivatization with trimethylsilyl (TMS) was performed to transform hydroxyl groups into more stable and volatile derivatives.

GC–MS analysis revealed different chemical profiles of *P. calyculatus* across hosts (*F. phillyreoides* and *Mimosa* sp.) and in all organs (pericarp, flower, leaf, and peduncle) and processing states (fresh and freeze-dried). The detected metabolites comprised organic acids, including oxalic acid, malic acid, citric acid, and ascorbic acid; phenolic acids, including gallic acid and pyrogallol; sugars, including arabinose, D-fructofuranose, and lactulose; polyols, including pinitol and myo-inositol; fatty acids, including palmitic acid and α-linolenic acid; sterols, including β-sitosterol; and triterpenes, including lupeol. As previously mentioned, these metabolites displayed clear variations depending on the host and organ type (Table 2).

The findings clearly demonstrated host- and organ-dependent variations in metabolite abundance, as indicated by the percentage of peak area. Gallic acid was predominant in the freeze-dried pericarp of *Mimosa* sp. (17.05%), yet it was not detected in *F. phillyreoides*. In the leaves, its concentration was 10.06% *in Mimosa* sp. compared to 1.40% (7-fold higher) in *F. phillyreoides*; and in flowers, it was 12.54% compared to 9.55% (31% higher). Ascorbic acid levels exhibited variability, with *Mimosa* sp. freeze-dried flowers (16.00%) surpassing those of *F. phillyreoides* (11.89%, 34% higher), while a contrasting trend was observed in pericarp (13.83% in *F. phillyreoides* vs. 10.55% in *Mimosa* sp.). It is evident that other compounds exhibited heightened specificity, with myo-inositol detected in *F. phillyreoides* flowers (3.96%); pinitol identified in the pericarp of *Mimosa* sp. (1.02%), and palmitic acid detected exclusively in *F. phillyreoides* (2.24%). Overall, the freeze-dried pericarp of *Mimosa* sp. accumulated the highest proportion of metabolites (97.70%), which is 2.5 times more than that of *F. phillyreoides* (39.12%). Among these, gallic acid was identified as one of the most abundant and widely distributed metabolites. Its quantification was confirmed using an analytical standard, as outlined in Table 3.

The variation in the concentration of gallic acid in *P. calyculatus* was also dependent on the host, plant organ, and processing state. Freeze-dried pericarp of *Mimosa* sp. reached 40.402 mg/g DW, exceeding freeze-dried pericarp from *F. phillyreoides* by 42.9%. In both hosts, the pericarp exhibited the highest concentrations of gallic acid compared to the flowers, leaves, and peduncle, representing more than 50% of the total gallic acid content in each case. In fresh organs, *Mimosa* sp. exhibited higher values compared to *F. phillyreoides*, with differences reaching up to 54.5% in the pericarp. These findings show a clear predominance of the pericarp, particularly in *Mimosa* sp., as the main reservoir of this phenolic compound.

### 2.3. Biological Activities

#### 2.3.1. Antioxidant Activity by HPTLC-DPPH· and HPTLC-ABTS·^+^ in Pericarp and Flower

**Figure 3:**
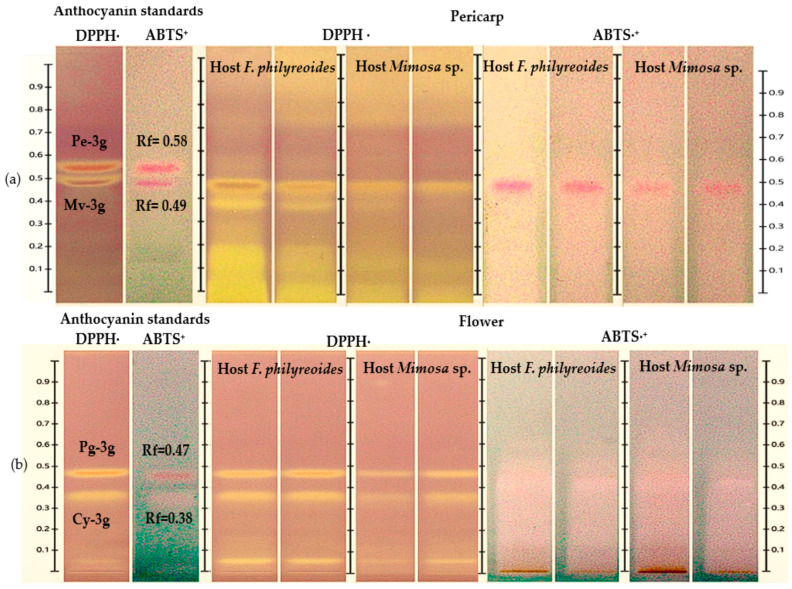
HPTLC chromatogram for the detection of antioxidant compounds in freeze-dried and fresh organs of pericarp (**a**) and flower (**b**) from methanolic extracts of *P. calyculatus*, obtained under white light after derivatization with the DPPH· and ABTS·^+^ reagents.

The HPTLC-DPPH· and HPTLC-ABTS^+^ analyses enabled the identification of antioxidant bands, as indicated by the distinctive color transitions (purple to yellow in DPPH· and blue to yellow-white in ABTS·^+^) (Figure 3a and Figure 3b, respectively). In the pericarp, bands with Rf = 0.49 (Mv-3g) were observed in both hosts and under both processing states (freeze-dried and fresh), with greater intensity in DPPH· and reduced response in ABTS^+^. In the case of flowers, bands with Rf = 0.47 (Pg-3g) were detected in both hosts, with stronger signals in DPPH· than in ABTS·^+^. This suggests a lower scavenging capacity against the ABTS·^+^ radical. It was observed that anthocyanins Pe-3g (pericarp) and Cy-3g (flower) exhibited subtle colorimetric shifts, likely attributable to their presence at trace levels, as evidenced by the low intensity noted in prior chromatograms. Consequently, they were solely regarded as detected compounds.

These results demonstrate that while both organs exhibited the presence of anthocyanins, which are known to possess antioxidant properties, the extent of this response and the intensity of the resulting signal varied depending on the specific host and the radical that was subjected to analysis. This suggests the potential for differences in the concentration or composition of these compounds.

#### 2.3.2. Antioxidant Activity by HPTLC-DPPH· and HPTLC-ABTS·^+^ in Leaf

**Figure 4:**
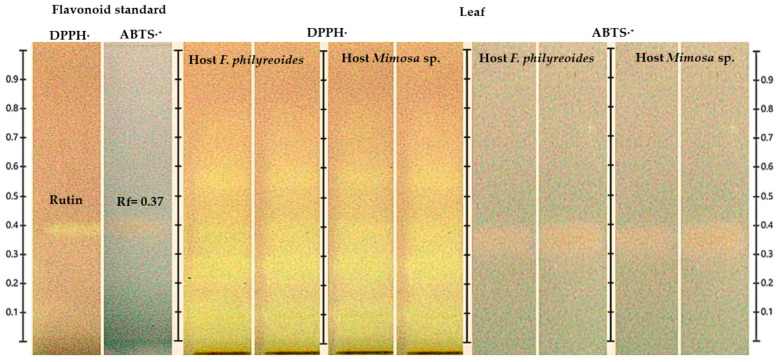
HPTLC chromatogram for the detection of antioxidant compounds in freeze-dried and fresh leaf organs from methanolic extracts of *P. calyculatus*, obtained under white light after derivatization with the DPPH· and ABTS·^+^ reagents.

The detection of the flavonoid in the leaf was confirmed by HPTLC coupled with DPPH· and ABTS·^+^ assays (Figure 4). The band corresponding to the rutin standard (Rf = 0.37) exhibited a characteristic color change in DPPH· (purple to yellow), indicative of a robust radical scavenging capacity. In contrast, the response observed in ABTS·^+^ was less pronounced, shifting from blue to yellow-white. The presence of signals consistent with rutin was observed in leaf samples of *P. calyculatus* parasitizing both *F. phillyreoides* and *Mimosa* sp., with greater discoloration intensity in DPPH· compared to ABTS·^+^. The observed discrepancy indicates a heightened efficacy in the neutralization of DPPH· radicals in comparison to ABTS·^+^ radicals.

#### 2.3.3. Quantification of Antioxidant Activity Using the DPPH· and ABTS·^+^ Techniques

A comprehensive evaluation of the two methodologies employed revealed that freeze-dried organs exhibited superior antioxidant activity in *P. calyculatus*, irrespective of the host. This observation was characterized by higher inhibition percentages, lower IC_50_ values, and higher Trolox equivalent concentrations.

The DPPH· assay (Table 4) was used to determine the maximum antioxidant capacity, which was observed to be 85.67% inhibition, IC_50_ = 50.50 μg/mL, and 486.56 μM TE/g DW, in the freeze-dried pericarp of *Mimosa* sp. The freeze-dried pericarp of *F. phillyreoides* followed with 83.11% inhibition, IC_50_ = 78.66 μg/mL, and 396.56 μM TE/g DW. The lowest activity was observed for fresh peduncle from *F. phillyreoides* (36.03% inhibition, IC50 = 261.44 μg/mL, 112.66 μM TE/g FW) and *Mimosa* sp. (36.89% inhibition, IC_50_ = 148.01 μg/mL, 191.48 μM TE/g FW).

In the ABTS·^+^ assay (Table 5), the freeze-dried pericarp of *Mimosa* sp. exhibited the highest activity (94.88% inhibition, IC_50_ = 106.31 μg/mL, 557.08 μM TE/g DW), followed by the freeze-dried pericarp of *F. phillyreoides* (89.76% inhibition, IC_50_ = 120.48 μg/mL, 523.13 μM TE/g DW). The lowest values were recorded in fresh peduncle from *F. phillyreoides* (41.72% inhibition, IC_50_ = 362.31 μg/mL, 128.04 μM TE/g FW) and *Mimosa* sp. (45.17% inhibition, IC_50_ = 238.08 μg/mL, 141.43 μM TE/g FW).

A comparative analysis revealed that ABTS·^+^ exhibited a more extensive inhibition range and higher Trolox equivalent values compared to DPPH·. This observation indicates that ABTS·^+^ is more sensitive to detecting disparities among high-activity organs. In both assays, freeze-dried pericarp was identified as the organ with the highest antioxidant capacity. In contrast, fresh peduncle was found to have the lowest antioxidant capacity. These findings are consistent with the observed patterns across different methods and hosts.

#### 2.3.4. Hypoglycemic Activity

The inhibitory activity against α-glucosidase also demonstrated significant variations both among hosts and among the organs of *P. calyculatus* (Table 6). In general, samples parasitizing *Mimosa* sp. exhibited higher activity than those from *F. phillyreoides*. Freeze-dried pericarp of *Mimosa* sp. exhibited 8.43% higher inhibition compared to freeze-dried pericarp of *F. phillyreoides* and presented the lowest IC_50_ value among all organs evaluated (85.53 μg/mL). Similarly, the analysis of freeze-dried *Mimosa* sp. flowers revealed the highest recorded activity (59.47% inhibition, IC_50_ = 84.07 μg/mL), which exceeded the activity observed in fresh flowers of *F. phillyreoides* (33.69% inhibition) by 76.55%.

With respect to the performance of the organ types, freeze-dried flowers and pericarp of *Mimosa* sp. were identified as the most active, with inhibition values above 58% and IC_50_ values below 86 μg/mL. In contrast, peduncle organs, especially fresh ones, exhibited the lowest inhibitory capacity in both hosts, with inhibition values ranging between 17.99% and 41.05% and IC_50_ values above 121 μg/mL. Regarding the leaves, they exhibited intermediate activity levels. In *F. phillyreoides*, no differences were observed between freeze-dried and fresh leaves. In the case of *Mimosa* sp., however, fresh leaves showed 33.42% lower activity compared to freeze-dried ones.

#### 2.3.5. Effect of Host, Organ, and Processing on *P. calyculatus*

**Figure 5:**
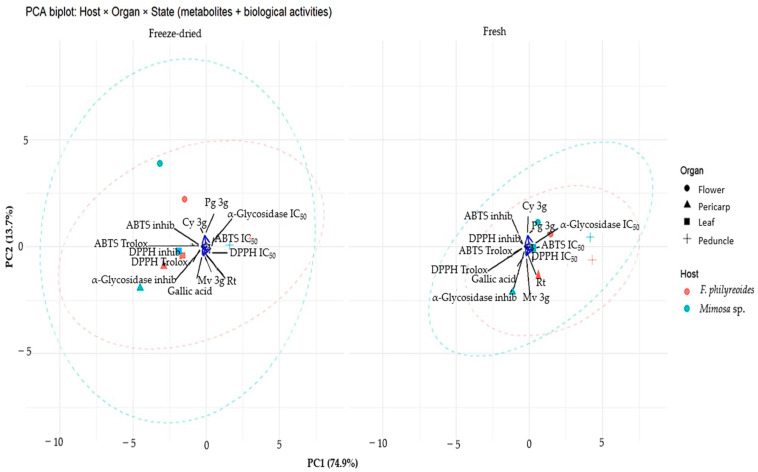
Principal Component Analysis (PCA) of *P. calyculatus* according to host, organ, and processing type (freeze-dried vs. fresh). Points represent observations (color = host; shape = organ), and dashed ellipses indicate the 95% confidence region for each host within each state. Arrows represent variable loadings: metabolites (gallic acid, Mv-3g, Pg-3g, Cy-3g, and rutin) and biological activities (DPPH· / ABTS·^+^ as Trolox equivalents, % inhibition and IC_50_; α-glucosidase inhibition and IC_50_). Projection of samples in the direction of DPPH· / ABTS·^+^ indicates higher antioxidant capacity (opposite direction = higher IC_50_ values). PC1 and PC2 explain 74.9% and 13.7% of the total variance, respectively.

In order to analyze the aforementioned effect, a principal component analysis (PCA) was performed (Figure 5). PC1 captured the primary gradient associated with antioxidant capacity: the DPPH·/ABTS·^+^ vectors (Trolox equivalents, % inhibition) were projected in the same direction, while their IC_50_ values were oriented in the opposite direction, indicating a negative correlation between antioxidant performance and IC_50_. PC2 exhibited a lesser degree of discrimination against hypoglycemic–α-glucosidase activity, with α-glucosidase IC_50_/inhibition contributing the most variation along this axis. Among the metabolites examined, gallic acid exhibited a direct correlation with the antioxidant assays, suggesting a primary contribution to the observed behavior. In contrast, Mv-3g, Pg-3g, Cy-3g, and rutin were positioned near the origin, indicating either more moderate or organ-specific effects.

A comparison of processing states revealed that freeze-dried organs exhibited greater dispersion and a more pronounced ellipsoidal separation between hosts. This finding suggests that processing may enhance chemical–functional differences. Conversely, fresh organs exhibited a tendency to congregate more proximally, indicating reduced variability. The orientation of the samples toward the direction of the Trolox (% inhibition vectors) was interpreted as indicative of higher radical-scavenging capacity. Conversely, the opposite positioning indicated higher IC_50_ values (lower potency). These results demonstrate that the bioactive profile of *P. calyculatus* is influenced by both freeze-drying and host modulation, with gallic acid as the predominant chemical marker and hypoglycemic–α-glucosidase activity explained mainly by the second component.

**Figure 6:**
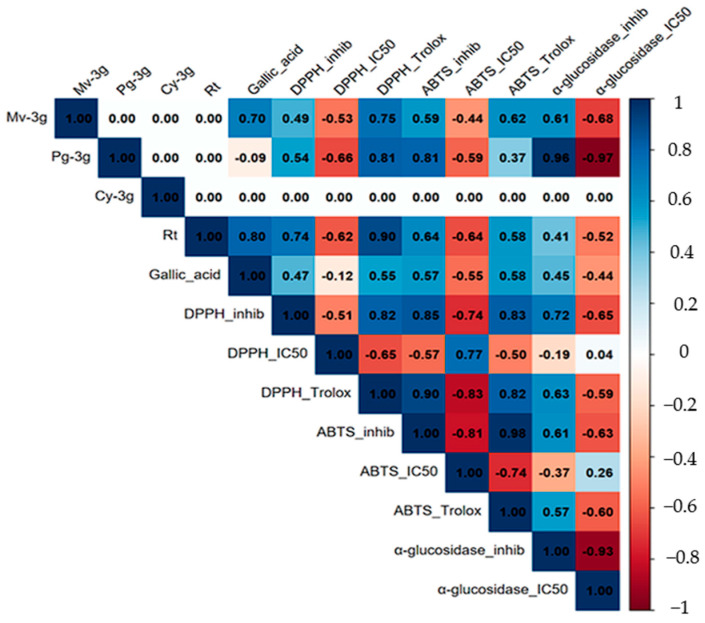
Pearson correlation matrix between metabolites (Mv-3g, Pg-3g, Cy-3g, rutin, and gallic acid) and biological activities (DPPH· and ABTS·^+^ as Trolox equivalents, % inhibition and IC_50_; α-glucosidase % inhibition and IC_50_). Correlation coefficients (r) are shown within each cell. Cells with “0.00” correspond to non-estimated pairs (insufficient data or zero variance) and should not be interpreted as absence of correlation.

To further analyze the correlation between metabolite content and observed biological activities, a Pearson correlation analysis was performed (Figure 6). This analysis revealed high internal coherence among the antioxidant assays. The trolox equivalents of DPPH· and ABTS·^+^ demonstrated a positive correlation with each other. Concurrently, both exhibited a negative association with their respective IC_50_ values. This finding is consistent with the hypothesis that an increase in antioxidant capacity is associated with lower IC_50_ (typical values, r = 0.6–0.9 for positive associations and r = −0.5 to −0.8 for negative associations). In a similar manner, hypoglycemic α-glucosidase activity exhibited a robust inverse relationship between percentage inhibition and IC_50_ (r = −0.93), thereby confirming the consistency between these indicators.

With respect to metabolites, rutin exhibited robust positive correlations with antioxidant signals, particularly with DPPH· Trolox equivalents, and negative correlations with IC_50_, indicating a substantial contribution to radical scavenging capacity. Gallic acid demonstrated moderate correlations with antioxidant responses and negative associations with IC_50_, thereby validating its role as a significant phenolic component in the observed bioactivity. In contrast, the anthocyanins (Mv-3g, Pg-3g, and Cy-3g) exhibited an incomplete pattern due to their restricted detection in specific organs or processing states (freeze-dried vs. fresh). Consequently, the estimation of several cells shown in Figure 6 was not possible, and they were indicated as “0.00.”. This should not be interpreted as an absence of correlation but rather as insufficient data for those pairs.

A collective examination of the results suggests a potential association between rutin and gallic acid with antioxidant activity. However, the contribution of anthocyanins remains to be validated with a more substantial sample size.

## 3. Discussion

The chemical variability observed in *P. calyculatus* is indicative of the influence of host, organ, and processing, a pattern that has also been documented in other mistletoe species. For instance, in *Viscum album* and *Phragmanthera incana*, the host identity has been demonstrated to influence not only the concentration of phenolics and flavonoids but also the range of biological activities [11,19,22,23,24]. In the present study, the preponderance of malvidin 3-glucoside in the pericarp and the presence of cyanidin 3-glucoside in the flowers of *Mimosa* sp. correspond to the earlier findings that the reproductive structures of mistletoes function as reservoirs of anthocyanins and phenolic compounds [15,18]. The findings indicate that the modulation of secondary metabolism in *P. calyculatus* by the host organism aligns with a prevalent pattern previously documented within the family Loranthaceae. This observation lends further credence to the ecological and pharmacological significance of host–parasite interactions.

In these species, several studies have documented significant variations in the content and type of secondary metabolites depending on the parasitized tree species and the plant organ analyzed. Such disparities have been ascribed to the impact of the host on the availability of nutrients, precursor metabolites, and physiological signaling. Additionally, the distinct metabolic response of mistletoe in each organ has been implicated, resulting in the generation of phytochemical profiles that are adapted to the unique conditions of each parasitic interaction [11,19,22,23,24].

In the GC-MS analysis, gallic acid was identified as one of the most prevalent phenolic acids, with its highest concentrations found in the freeze-dried pericarp of *Mimosa* sp. (17.05%), and significantly higher proportions in its leaves compared with those of *F. phillyreoides*. This finding corroborates its established role as an antioxidant and anticancer metabolite, as previously reported in *V. album* [20]. The detection of β-sitosterol and lupeol serves to further substantiate the phytochemical similarity with other mistletoes, in which sterols and triterpenes, including oleanolic and ursolic acids, have been associated with anti-inflammatory and immunomodulatory properties [21,25].

The exclusive identification of pinitol in the pericarp of *Mimosa* sp. is consistent with its function as an osmoprotectant described in *V. album* and other species [26]. Meanwhile, myo-inositol, detected primarily in *F. phillyreoides* flowers, complements this polyol profile, suggesting a potential role in osmotic adjustment. Furthermore, the presence of D-fructofuranose, arabinose, and lactulose suggests the existence of a repertoire of structural and reserve carbohydrates that may be pertinent to osmotic regulation and cell integrity [26,27,28].

Quantitatively, the freeze-dried pericarp of *Mimosa* sp. exhibited the highest overall proportion of detected compounds (97.70%), which was 2.5-fold higher than in *F. phillyreoides* (39.12%). This pattern lends support to the hypothesis that metabolite accumulation in mistletoes is organ- and host-dependent. In the same way, it is consistent with reports in *V. album* attributing antioxidant variability to differences in phenolic, triterpenic, and organic acid content depending on tissue type and processing [19].

Even though lectins and viscotoxins have been shown to possess immunomodulatory and anticancer properties, these compounds were not detected by GC–MS due to their macromolecular nature. However, their presence in mistletoes has been extensively validated through proteomic studies [29]. Taken together, these results demonstrate that *P. calyculatus* possesses a multifunctional and host-influenced phytochemical composition that contributes to its bioactivity and ecological adaptability. To date, no reports have analyzed this species parasitizing *Mimosa* sp. and *Forestiera* sp. Therefore, this study is pioneering in its documentation of host-dependent chemical variation in *P. calyculatus*.

The results obtained with DPPH· and ABTS·^+^ confirmed that the antioxidant capacity of *P. calyculatus* was modulated by host, organ, and processing. This is consistent with reports on this species and other Loranthaceae, such as *Phragmanthera incana*, *P. capitata*, and *Cladocolea loniceroides*. In these species, variation in phenolic content and antioxidant activity has been documented according to host species and plant organ evaluated [9,22,30,31].

In this study, the freeze-dried pericarp of *Mimosa* sp. exhibited the highest antioxidant activity, with 85.67% DPPH· and 94.88% ABTS·^+^ scavenging capacity. It demonstrated low IC_50_ values and high Trolox equivalents, surpassing the values of the freeze-dried pericarp of *F. phillyreoides* by 3.08% (DPPH·) and 5.12% (ABTS·^+^), respectively. This is consistent with the findings described by Ochoa et al. [15], who reported that fruits of *P. calyculatus* parasitizing *Prosopis laevigata* (Fabaceae) exhibited 21.4% higher total phenols and 18.6% higher ABTS·^+^ activity compared to those parasitizing *Quercus deserticola* (Fagaceae). In a similar vein, Flores et al. [32] discovered that the flowers and leaves of *P. calyculatus* contain elevated levels of phenols and flavonoids, accompanied by low IC_50_ values in DPPH·. They also observed that reproductive structures exhibit higher levels of activity compared to support structures, a finding that aligns with the superiority of the pericarp as evidenced in this study.

As demonstrated by Reynoso et al. [18], the substantial polyphenol content (73.54 mg GAE/g) and flavonoid content (39.37 mg CE/g) present in freeze-dried aqueous extracts of *P. calyculatus* leaves appear to be associated with a protective effect against genetic damage. This protective effect is believed to result from the neutralization of reactive species and the stimulation of cellular antioxidant systems. Although radical scavenging capacity was not evaluated in that study, the anti-genotoxic activity observed therein coincides with the pattern recorded here. Specifically, organs with higher phenolic concentration, particularly freeze-dried pericarp, demonstrated the highest antioxidant capacity.

In *Cladocolea loniceroides*, Serrano et al. [30] reported 83.7% DPPH· inhibition and an IC_50_ of 3.04 mg eq. The concentration of the substance in question, expressed in milligrams per liter (mg/L), reached 82.6% in methanolic fruit extract, with a median effective concentration (EC_50_) of 0.39 milligrams equivalent (mg eq). The values of GA/g in aqueous extract are indicative of elevated levels of polyphenols and flavonoids. The observed phenomenon of higher bioactive metabolite concentrations in fruits compared to stems or peduncles aligns with the previously documented trend in *P. calyculatus*.

In *V. album*, it has been demonstrated that the host significantly influences the phenolic profile and antioxidant capacity. The leaves that parasitized *Populus alba* exhibited elevated levels of flavonols and had the most significant antioxidant activity in comparison to other hosts, as evaluated by DPPH· and FRAP. The authors concluded that the composition and antioxidant activity of mistletoe are contingent on the host tree [33]. This observation is consistent with the results of the present study, wherein *Mimosa* sp. exhibited consistently higher inhibition percentages than *F. phillyreoides* (3.08% higher in DPPH· and 5.12% higher in ABTS·^+^). Additionally, *Mimosa* sp. demonstrated lower IC_50_ values and higher Trolox equivalents.

As Nicoletti’s review indicates [34], a variety of mistletoes, including *V. album*, as well as other genera such as Taxillus, Ligaria, Tristerix, and Psittacanthus, have been evaluated by DPPH· and ABTS·^+^. The activity of these mistletoes has been attributed to flavonoids, phenolic compounds, and triterpenes (e.g., gallic acid, oleanolic acid, and lupeol). The evidence supports the interpretation of the results obtained in this study, which point to a diverse phytochemical profile, which is sensitive both to the organ evaluated and to the processing state. This profile highlights the tendency of freeze-dried organs to exhibit greater antioxidant capacity.

In the study conducted by Hlophe and Bassey [35], the presence of antioxidant bands was demonstrated in *Loranthus micranthus* through TLC bioautography and 2,2-diphenyl-1-picrylhydrazyl (DPPH·) assay. These antioxidant bands were found to be associated with flavonoids, tannins, and terpenoids. Furthermore, marked differences in antioxidant activity were documented among extracts of different geographic origin, thereby confirming that geographic variation and host significantly modify antioxidant potency. This behavior is analogous to the superiority exhibited by *Mimosa* sp. in this study in comparison to *F. phillyreoides*, indicating that the host-dependent pattern is a prevalent phenomenon within the Loranthaceae family.

A comprehensive review of the extant literature suggests that mistletoes exhibit high antioxidant capacity due to their richness in phenols, flavonoids, anthocyanins, and triterpenes. The magnitude of the effect is determined by the host and organ, and controlled dehydration (freeze-drying) concentrates bioactive metabolites. For *V. album*, for instance, leaves and fruits that have adapted to parasitizing different tree species exhibited significant variations in phenols, flavonoids, and antioxidant activity (DPPH·, ABTS·^+^, and FRAP). The maximum values were observed in fruits and in hosts such as *Betula pendula* and *Acer platanoides* [23,36].

Research conducted by Majeed et al. and Mapfumari et al. [19,37,38] has demonstrated that in miscellaneous species of mistletoe, such as *V. album* and *V. continuum*, the antioxidant activity is influenced by both the host organism and the extraction solvent utilized. In *V. album*, the highest concentrations of phenols (13.46 ± 0.87 mg GAE/g), flavonoids (2.38 ± 0.04 mg RE/g), and antioxidant capacity by FRAP (500.63 ± 12.58 µM Fe^2+^/g DW) were recorded in leaves parasitizing *Juglans regia* and extracted with ethanol, exceeding those from *Populus ciliata* and *Ulmus villosa*. In *V. continuum*, the greatest inhibition was obtained in methanolic extract (98% in DPPH· and H_2_O_2_) with an IC_50_ of 0.11 mg/mL, followed by acetone (79%), hexane (63%), and dichloromethane (45%). The results suggest that both solvent polarity and host effects influence antioxidant activity, thereby affecting the bioactive profile [19,37]. The present study’s findings align with the aforementioned pattern, wherein the freeze-dried pericarp of *Mimosa* sp., a specimen notable for its high phenol content and solubility in polar solvents, exhibited the most pronounced inhibitory effects (85.67% DPPH; 94.88% ABTS·^+^) and the lowest IC_50_ values. This specimen’s efficacy surpassed that of the pericarp of *F. phillyreoides* by 3.08% (DPPH·) and 5.12% (ABTS·^+^), a discrepancy that can be further elucidated by the substantial influence of host and solvent on the antioxidant capacity of *Psittacanthus* and *Viscum* species, as reported in previous studies [4,15,18,34,35].

The evidence presented herein indicates that the peak activity recorded in the freeze-dried pericarp of *Mimosa* sp. and the nadir in the fresh peduncle of both hosts align with a prevailing pattern observed in the families Loranthaceae and Santalaceae. This pattern suggests that bioactivity is maximized in reproductive organs and under conditions that concentrate active metabolites [1,2,16].

In addition to phytochemical characterization and antioxidant evaluation, the antihyperglycemic activity of *P. calyculatus* was determined through α-glucosidase inhibition in different organs and hosts. The findings indicated that this activity was also influenced by the host, the organ type, and the processing state. The highest recorded inhibition values and the lowest IC_50_ values were observed in freeze-dried organs of *Mimosa* sp., specifically flowers and pericarp. This pattern indicates that the process of dehydration has the capacity to enhance the concentration or stability of bioactive metabolites that are responsible for the inhibition of enzymes [8,16].

These findings are consistent with those reported by Ávila et al. [8], who demonstrated that methanolic extract of *P. calyculatus* significantly reduced glycemia in streptozotoc-induced diabetic rats, both in acute treatments and in chronic regimens, without genotoxic effects in the micronucleus assay. The study identified condensed tannins, (+)-catechin, and gallic acid as the primary active compounds, which is consistent with the findings of our analysis. The results support the hypothesis that these compounds contribute to the observed inhibitory activity.

A parallel set of findings has been documented in the scientific literature concerning a different species belonging to the Loranthaceae family. In the study by Noman et al. [17], it was reported that the crude extract and chloroform fraction of *Loranthus acaciae* led to a reduction in glucose levels of up to 47% in diabetic rats. This effect was attributed to the presence of flavonoids, including quercetin-3-O-β-D-glucopyranoside, quercetin-3-O-β-(6-O-galloyl)-glucopyranoside, and catechin-7-O-gallate. The aforementioned metabolites have been associated with the inhibition of both α-glucosidase and α-amylase, in addition to the modulation of enzymes that are involved in the maintenance of glucose homeostasis.

In a similar manner, significant reductions in glycemia have been documented in rats treated with ethanolic leaf extracts of *Dendrophthoe falcata*, another hemiparasitic mistletoe of the same family. These findings serve to reinforce the antihyperglycemic potential of the group [39]. The existing body of evidence suggests a potential relationship between the hypoglycemic effect of *P. calyculatus* and the synergism among polyphenols (catechin, gallic acid), flavonoids, and triterpenes. These compounds have been previously identified in the species and may act by modulating carbohydrate-digesting enzymes and metabolic pathways associated with glucose regulation.

The in vitro results obtained from this study align with the existing in vivo evidence, thereby supporting the hypothesis that the most active organs, particularly those derived from freeze-dried flowers and pericarp of *Mimosa* sp., serve as a concentrated source of bioactive compounds that possess therapeutic potential in the management of type 2 diabetes. Further studies are deemed necessary to comprehensively characterize the responsible metabolites and evaluate their efficacy and safety profiles in animal models.

## 4. Materials and Methods

### 4.1. Reagents

Methanol, DPPH· (1,1-diphenyl 2-picrylhidrazyl), ABTS·^+^ (2,2′-azino-bis (3-ethylbenzothiazoline-6-sulfonic acid) diammonium salt), Trolox ((±)-6-Hydroxy-2,5,7,8-tetramethylchromane-2-carboxylic acid), natural products (NP reagent, 2-amino ethyl diphenyl borinate); phenolic compounds, flavonoids and anthocyanins standards: gallic acid (GA), rutina (Rt), malvidin 3-glucoside (Mv-3g), Peonidin 3-glucoside (Pe-3g), Pelargonidin 3-glucoside (Pg-3g) and Cyanidin 3-glucoside (Cy-3g); C8–C40 alkanes calibration standard, dimethyl sulfoxide (DMSO), α-glucosidase enzyme, p-nitrophenyl glucopyranoside (P-NPG), and acarbose were purchased from Sigma-Aldrich^®^ (St. Louis, MO, USA). The silica gel chromatographic plates 60 F_254_ (20 cm × 10 cm, Art. 1.05729.0001), used in the HPTLC technique, were supplied by Merck (Darmstadst, Hesse, Germany). The solvents used for the mobile phases, ethyl acetate, acetic acid and formic acid were obtained from Sigma-Aldrich^®^ (St. Louis, MO, USA). Pyridine (ACS grade, ≥99% purity; Karal^®^, Mexico City, Mexico) was used for GC-MS derivatization. Trimethylchlorosilane (Sigma-Aldrich, St. Louis, MO, USA). Isoctane (2,2,4-trimethylpentane) of 99% purity was obtained from Sigma-Aldrich (St. Louis, MO, USA).

### 4.2. Plant Samples

The organs of *P. calyculatus* were collected from two host species, *F. phillyreoides* and *Mimosa* sp., in the locality of Los Tábanos, Jiquilpan, Michoacán, Mexico. The geographical coordinates for the sampled hosts were 19°59′4″ N, 102°41′39″ W (*F. phillyreoides*) and 19°59′3″ N, 102°41′26″ W (*Mimosa* sp.). The collections were carried out in July 2024 and May 2025. The botanical identification of parasitic plants was performed by M.S. Ignacio García Ruíz, and voucher specimens were deposited in the CIIDIR–Michoacán Herbarium (CIMI) under accession numbers 13693 (*P. calyculatus* on *F. phillyreoides*) and 13694 (*P. calyculatus* on *Mimosa* sp.).

### 4.3. Sample Conditioning

The organs were separated and washed with distilled water. Subsequently, the pericarp, seed, and viscin were separated from the fruit, frozen in liquid nitrogen, and freeze-dried for preservation (FreeZone 12 Liter, LABCONCO, LABCONCO Corporation, Kansas City, MO, USA). Organs were prepared as freeze-dried and fresh samples. In the case of fresh organs, the freeze-drying process was omitted; instead, they were ground with liquid nitrogen using a mortar [15].

### 4.4. Extraction of Samples

Both fresh and freeze-dried organs were used for extract preparation. Fresh organs were immediately frozen with liquid nitrogen and ground to a fine powder, and 30 mg of homogenized material was accurately weighed. For freeze-dried tissue, an equivalent portion of the same homogenized material was lyophilized prior to weighing (FreeZone 12, LABCONCO^®^, Kansas City, MO, USA). The results were deliberately expressed as fresh weight for non-lyophilized samples and as dry weight for freeze-dried tissues to emphasize the impact of processing on the concentration of phenolic compounds and the enhancement of biological activities. Acidified methanolic extract (HCl 1 N, 85:15 *v*/*v*) was prepared as extraction solvent. Pericarp, leaf, flower, and peduncle (30 mg) were placed in microtubes, 1 mL of solvent was added, and samples were sonicated (60 Hz/30 min; ultrasonic bath PNKKODW, Rohs^®^, CIN, Sunnyvale, CA, USA) at room temperature (25 ± 2 °C). Subsequently, samples were centrifuged at 3500 rpm for 10 min (Hermle Labortechnik GmbH, Z 233 M-2, Wehingen, Baden-Württemberg, Germany), filtered through a 0.22 μm nylon membrane (Millipore^®^, Jaffrey, NH, USA), and concentrated in a rotary evaporator (R-II, BUCHI^®^, Zurich, Switzerland). Extracts of fresh organs were freeze-dried for further analysis [15].

### 4.5. Detection and Quantification of Anthocyanins and Flavonoid Compounds by HPTLC

Detection of anthocyanins, phenolic compounds, and flavonoids was performed by high-performance thin-layer chromatography (HPTLC) using the method described by Creţu et al. [40] with modifications. Acidified methanolic extracts obtained as described in Section 4.4 (filtered through 0.22 μm nylon membranes) were directly applied to HPTLC silica gel plates for separation and quantification of anthocyanins and flavonoids. The samples (30 mg/mL) and standards of gallic acid, Rt, Mv-3g, Pe-3g, Pg-3g, and Cy-3g (100 μg/mL in MeOH) were applied using an Automatic TLC Sampler 4 (ATS4, CAMAG^®^, Muttenz, Switzerland) on 20 × 10 cm plates, with a band spacing of 10 mm, a distance of 8 mm from the lower edge, and 15 mm from the left side; 18 bands were applied. Acidified methanolic extracts (30 mg/mL) of pericarp, flowers, leaves, and peduncles of *P. calyculatus* were examined in triplicate (150 nL/s).

The plate was developed in an Automated Developing Chamber 2 (ATS4, CAMAG^®^, Muttenz, Switzerland) at a relative humidity of 47 ± 2% (humidity controller with saturated potassium thiocyanate solution), using a mobile phase consisting of toluene, ethyl acetate, formic acid, and water (10:1.1:1.1:2.3, *v*/*v*/*v*/*v*). The migration distance was 70 mm, and the development time was 34 min. After development, the plate was dried with cold air for 5 min. Following chromatographic separation, the plate was heated on a TLC Plate Heater III (ATS4, CAMAG^®^, Muttenz, Switzerland) at 100 °C for 5 min and derivatized using a TLC Immersion Device III (ATS4, CAMAG^®^, Muttenz, Switzerland) at a vertical speed of 3 cm/s, with a 1% Natural Products (NP) solution to reveal anthocyanins and flavonoids (1 g diphenylboric acid 2-aminoethyl ester diluted in 100 mL methanol); immersion time was 3 s. After derivatization, the plate was reheated (3 min, 100 °C) to remove excess solvent. Plates were evaluated using a TLC Visualizer Documentation System (CAMAG^®^). All images were captured under white light and UV light at 366 nm. Data were processed with VisionCats software (CAMAG^®^) version 2.4. Mv-3g, Pg-3g, Cy-3g, and Rt were quantified by estimating peak heights using calibration curves (Mv-3g = −3.27 × 10^−14^x^2^ + 2.159 × 10^−7^x − 4.039 × 10^−2^, R^2^ = 0.9997, Rf = 0.49; Pg-3g = (4.863 × 10^−7^x/1.852 × 10^−6^ + x) + (2.882 × 10^−4^), R^2^ = 0.9999, Rf = 0.47; Cy-3g = 6.808 × 10^−8^x − 1.391 × 10^−2^, R^2^ = 0.9986, Rf = 0.38; Rt = (1.132 × 10^−1^x/3.919 × 10^−8^ + x) − (5.998 × 10^−2^), R^2^ = 0.9542). Different volumes (0.1, 0.3, 0.5, 0.7, and 0.9 μL, equivalent to 10, 30, 50, 70, and 90 μg, respectively) of the standard solutions were spotted in triplicate on the plates.

#### Volatile Compounds Identification by GC–MS

From acidified methanolic extracts of pericarp, flower, leaf, and peduncle of *P. calyculatus* at a concentration of 30 mg/mL, 50 µL was taken and placed in Eppendorf tubes. For sample concentration under vacuum, an Eppendorf Concentrator plus/Vacufuge^®^ plus (Eppendorf, Hamburg, Germany) was used at 45 °C with a vacuum level of 20 mbar. The equipment included a maintenance-free diaphragm pump and a coated lid providing chemical resistance against acids and organic solvents.

Since phenolic and flavonoid compounds of *P. calyculatus* are non-volatile and thermolabile, a derivatization step was required prior to gas chromatography–mass spectrometry analysis. Therefore, *P. calyculatus* extracts and the gallic acid standard were derivatized by adding 20 μL of pyridine and 100 μL of N,O-bis(trimethylsilyl)trifluoroacetamide (BSTFA), which converted hydroxyl groups into more volatile and stable trimethylsilyl derivatives suitable for GC–MS detection.

Samples of *P. calyculatus* and gallic acid standard were processed by reaction with the addition of 20 μL pyridine and 100 μL N, O-bis(trimethylsilyl)trifluoroacetamide (BSTFA). The mixture was incubated for 30 min at 80 °C in a Thermomixer Comfort (Eppendorf, Hamburg, Germany) to ensure homogeneous mixing and constant temperature. Finally, 100 µL iso-octane was added, and each sample was injected into the GC–MS system (Clarus 680/Clarus SQ 8T, Perkin-Elmer^®^, Waltham, MA, USA) equipped with an Elite 5MS capillary column (30 m × 0.25 mm I.D. × 0.25 μm film thickness).

The analysis was carried out according to Quintana et al. [16] under the following conditions: oven initial temperature of 70 °C for 5 min, followed by an increase of 5 °C/min up to 280 °C, held for 15 min. The autosampler injector operated at 250 °C, with a split ratio of 20:1 and a solvent delay of 5 min. Helium was used as carrier gas at a flow rate of 1 mL/min. The mass spectrometer was operated at 70 eV ionization voltage, with an interface temperature of 250 °C and a source temperature of 230 °C, in full-scan mode, with a mass range of 50–800 *m*/*z*. A capillary column of 30.0 m × 320 μm was employed.

Identification of compounds was carried out by comparing the calculated retention index (Ri) based on a homologous series of n-alkanes, the mass spectrum, and retention time (Rt), as well as through comparison with the NIST/EPA/NIH spectral database (2017). Results were expressed as relative areas of each peak (% area). Quantification of gallic acid was performed by estimating the area of the corresponding peaks using a 7-point calibration curve (0, 0.01, 0.02, 0.05, 0.09, 0.11, 0.18, and 0.22 µg/µL). Results were expressed as mg equivalents per g of freeze-dried and fresh weight (mg/g DW-FW) using the equation y = 5 × 109x − 9 × 107, R2 = 0.97.

### 4.6. In Vitro Biological Activities

#### 4.6.1. Antioxidant Activity by DPPH· and ABTS·^+^ Microdilution Method

For the DPPH· and ABTS·^+^ assays, as described by Bernal et al. [41] with some modifications, acidified methanolic extracts obtained as described in Section 4.2 were used. A volume of 20 μL of extract was mixed with 200 μL of DPPH· solution (150 μM) and incubated in darkness for 30 min. The decrease in absorbance was recorded at 515 nm using a spectrophotometer (SmartReader™ 96-T, MR9600-T, Accuris Instruments, Benchmark Scientific Inc., Edison, NJ, USA). Quantification was performed using a Trolox calibration curve (μM TE = −0.645[A_515_ nm] + 0.245, R^2^ = 0.984), and results were expressed as μM Trolox equivalents per gram of freeze-dried and fresh weight (μM TE/g DW-FW). Each sample was analyzed in triplicate, with five concentrations (0–0.60 μmol Trolox).

For the ABTS·^+^ assay, the radical was generated by oxidation with potassium persulfate and incubated for 24 h. Subsequently, the absorbance was adjusted with distilled water to 0.760 ± 0.001, using the same spectrophotometer. A volume of 20 μL of methanolic extract was added to 280 μL of the ABTS·^+^ solution, and the mixture was kept in darkness for 6 min. Absorbance of samples and blanks (methanol) was measured at 734 nm. Antioxidant capacity was expressed as μmol Trolox equivalents per gram of freeze-dried and fresh weight (μmol TE/g DW-FW) using a calibration curve (μmol TE = −0.94159[A_734_ nm] + 0.828, R^2^ = 0.997) with five concentrations (0–0.60 μmol Trolox).

IC_50_ values were determined from the response of DPPH· and ABTS·^+^ at four extract concentrations (0, 100, 300, 600, 800 μg/mL), and the inhibition percentage (100 μg/mL) was calculated using Equation (1).

(1) $$Inhibition\left(\%\right)=1-\frac{Sample\;absorbance}{Blank\;absorbance}\times 100$$

#### 4.6.2. Antioxidant Activity by HPTLC-DPPH and HPTLC- ABTS·^+^

HPTLC plates were subjected to the same conditions as described in Section 4.3. They were derivatized with methanolic DPPH· and ABTS·^+^ solutions to detect antioxidant activity of the separated bands [42].

#### 4.6.3. Hypoglycemic Activity by α-Glucosidase Inhibition

The inhibition of α-glucosidase was determined using the spectrophotometric microplate method with modifications according to Cárdenas et al. [43]. Extracts were dissolved in DMSO at 50% to obtain the working solutions, which were further diluted to five final concentrations (0, 100, 300, 400, and 600 μg/mL).

A total of 170 μL phosphate buffer (5 mM, pH 6.8), 10 μL of sample, and 10 μL of glucosidase (0.4 U/mL) were placed in a microplate and incubated for 10 min at 37 °C. Then, 10 μL of substrate (p-NPG, 0.5 mM) was added to the wells and incubated at 37 °C for 30 min. Absorbance was read at 405 nm (SmartReader™ 96-T, MR9600-T, Accuris Instruments, Benchmark Scientific Inc., Edison, NJ, USA). DMSO was used as negative control and acarbose (5 mg/mL) as positive control. IC_50_ was determined from α-glucosidase responses at four extract concentrations (0, 100, 300, 400, and 600 μg/mL). The percentage of inhibition (100 μg/mL) was calculated using Equation (1).

### 4.7. Statistical Analysis

Each experiment was performed in triplicate, resulting in a total of nine replicates per condition. A three-way analysis of variance (ANOVA) was conducted, and means were separated using Tukey’s test (*p* ≤ 0.05). The half-maximal inhibitory concentration (IC50) was calculated with Quest Graph™ IC50 Calculator, AAT Bioquest online software (https://www.aatbio.com/tools/ic50-calculator, accessed on 25 July 2025) [44]. Pearson’s correlation tests and principal component analysis (PCA) were carried out using R^®^ statistical software for Windows, version 4.4.2, 2025.

## 5. Conclusions

The present study underscores the existence of both host- and organ-dependent disparities in the phytochemical profile and biological activities of *Psittacanthus calyculatus* (Mexican mistletoe). Freeze-dried extracts demonstrated higher concentrations of phenolic compounds, particularly gallic acid, and anthocyanins, including malvidin-3-O-glucoside, pelargonidin-3-O-glucoside, and cyanidin-3-O-glucoside, in comparison to fresh tissues. Among the organs examined, the pericarp and flowers of *P. calyculatus* parasitizing *Mimosa* sp. hosts exhibited the highest antioxidant and α-glucosidase inhibitory activities, while fresh peduncles demonstrated the lowest values. The enhanced metabolite concentration and bioactivity observed in freeze-dried samples suggest that dehydration processes may favor the accumulation or stability of bioactive compounds. The findings indicate a correlation between the presence of specific metabolites (i.e., gallic acid, rutin, and anthocyanins) and the strong antioxidant and hypoglycemic potential observed in reproductive organs. Collectively, these findings substantiate *P. calyculatus* as a promising nutraceutical source with therapeutic relevance, particularly for managing oxidative stress and hyperglycemia. Nevertheless, further research is necessary to evaluate its efficacy in vivo, identify the mechanisms involved, and isolate the active compounds responsible for the observed effects. This will ensure the sustainable and safe exploitation of these hemiparasitic species.

## Figures and Tables

**Table 1:** Quantification of metabolites identified by HPTLC from different freeze-dried and fresh organs of *P. calyculatus*.

Host (H)	Plant Organ (O)	Metabolite	Type of Process (P)	Quantification mg/g FW-DW
*F. phillyreoides*	Pericarp	Mv-3g	Fresh	16.29 ± 0.234 ^fg^
			Freeze-dried	14.82 ± 60.283 ^f^
	Flower	Pg-3g	Fresh	2.33 ± 0.127 ^b^
			Freeze-dried	2.917 ± 0.293 ^bc^
		Cy-3g	Fresh	Traces
			Freeze-dried	18.597 ± 0.829 ^h^
	Leaf	Rt	Fresh	3.902 ± 0.254 ^bcd^
			Freeze-dried	4.901 ± 0.597 ^cd^
*Mimosa* sp.	Pericarp	Mv-3g	Fresh	17.206 ± 0.411 ^gh^
			Freeze-dried	27.433 ± 1.887 ^a^
	Flower	Pg-3g	Fresh	4.224 ± 0.556 ^bcd^
			Freeze-dried	7.14 ± 0.495 ^e^
		Cy-3g	Fresh	Traces
			Freeze-dried	26.557 ± 1.192 ^a^
	Leaf	Rt	Fresh	3.577 ± 0.296 ^bc^
			Freeze-dried	5.387 ± 1.248 ^de^
Interaction	H	***		
	O	***		
	P	***		
	H:O:P	***		

A three-way analysis of variance (ANOVA) was performed. *** indicates highly significant effects of the factors and their interaction (*p* < 0.001). The mean separation of the H:O:P interaction was performed using Tukey’s test (*n* = 9, *p* < 0.05). Different lowercase letters indicate significant differences among means.

**Table 2:** Differential metabolites detected by GC–MS in *P. calyculatus* across hosts, organs, and processing (fresh and freeze-dried).

Peak No.	Rt (min)	Ri ^1^	Compound Name	Organ and Peak Area %															
				*F. phillyreoides*								*Mimosa* sp.							
				Pericarp		Flower		Leaf		Peduncle		Pericarp		Flower		Leaf		Peduncle	
				F	FD	F	FD	F	FD	F	FD	F	FD	F	FD	F	FD	F	FD
1	8.166	1120	Oxalic acid	-	-	-	-	-	-	2.392	-	-	-	-	-	-	-	-	-
2	12.521	1261	Glycerol	-	-	-	-	-	-	-	-	-	1.577	-	-	-	-	-	-
3	18.22	1476	Malic acid	-	-	-	-	0.466	0.966	-	-	-	-	-	-	-	-	-	-
4	21.193	1605	Arabinose	-	-	-	-	-	-	-	-	-	1.72	-	-	-	-	-	-
5	25.234	1799	D-Fructofuranose	-	-	-	-	-	-	-	-	-	4.26	-	-	-	-	-	-
6	25.561	1814	Citric acid	-	-	-	-	-	-	-	-	-	1.556	-	-	-	-	-	-
7	25.691	1820	Pinitol	-	-	-	-	-	-	-	-	-	1.025	-	-	-	-	-	-
8	28.031	1936	**Ascorbic acid**	13.84	-	-	-	-	-	-	-	-	15.558	10.56	18.625	-	-	-	-
9	28.254	1948	**Gallic acid**	-	-	9.554	-	0.406	1.406	-	1.082	12.5	18.65	-	12.544	0.562	15.062	-	1.082
10	29.924	2038	Palmitic acid	-	2.239	-	-	-	-	-	-	-	-	-	-	-	-	-	-
11	30.237	2056	Myo-Inositol	0.15	0.081	-	3.965	-	0.556	-	-	-	-	-	-	-	-	-	-
12	32.883	2212	α-Linolenic acid	-	0.721	-	-	-	-	-	-	2.65	8.561	-	8.561	-	-	-	-
13	33.397	2243	Stearic acid	-	-	-	-	-	-	-	-	-	-	-	8.561	-	-	-	-
14	34.449	2307	Aucubin	-	-	-	-	-	-	-	-	-	-	-	2.572	-	-	-	-
15	38.888	2430	Monopalmitin	-	-	-	-	-	-	-	-	-	-	-	-	-	-	-	0.841
16	42.117	2464	Lactulose	-	-	-	-	-	-	-	-	-	-	-	0.555	-	-	-	-
17	49.33	2727	Lupeol	-	-	-	0.589	-	-	-	-	-	-	-	1.259	-	-	-	-
Total (%)				39.123								97.699							

^1^ Retention index relative to the C8–C40 alkane calibration standard. F = Fresh, FD = Freeze-dried. (-) = compound not detected. Compounds in bold are considered major compounds. Compounds derived from the derivatization agent (TMS) were excluded from the analysis and interpretation of the results, as they do not correspond to natural metabolites of *P. calyculatus*. Gallic acid was prioritized due to its abundance, biological relevance, and verification with an analytical standard.

**Table 3:** Quantification of gallic acid detected by GC-MS in freeze-dried and fresh *P. calyculatus* organs collected from two host species.

Host	Type of Process	Plant Organ	Gallic Acid mg/g DW-FW
*F. phillyreoides*	Fresh	Pericarp	17.311 ± 0.429 ^f^
	Freeze-dried		23.056 ± 0.097 ^g^
	Fresh	Flower	4.126 ± 0.200 ^a^
	Freeze-dried		13.312 ± 0.288 ^b^
	Fresh	Leaf	5.189 ± 0.189 ^c^
	Freeze-dried		12.46 ± 0.480 ^d^
	Fresh	Peduncle	ND
	Freeze-dried		5.129 ± 0.337 ^e^
*Mimosa* sp.	Fresh	Pericarp	38.153 ± 0.742 ^e^
	Freeze-dried		40.402 ± 0.228 ^f^
	Fresh	Flower	6.811 ± 0.574 ^a^
	Freeze-dried		7.138 ± 0.375 ^a^
	Fresh	Leaf	10.297 ± 0.430 ^b^
	Freeze-dried		16.204 ± 0.877 ^c^
	Fresh	Peduncle	ND
	Freeze-dried		4.2370 ± 0.417 ^d^

Mean ± standard deviation is shown (mg/g DW-FW). Tukey’s test was performed (*n* = 9, *p* < 0.05); different letters indicate significant differences in columns. Calibration curve: Y = 5.00 × 10^9^ *X* − 9.00 × 10^7^, R^2^ = 0.9705. ND = compound not detected.

**Table 4:** Antioxidant activity of freeze-dried and fresh organs of *P. calyculatus* using the DPPH· technique.

Host	Type of Process	Plant Organ	Inhibition % ^1^	IC_50_ μg/mL	DPPH• (μM TE/g DW-FW)
*F. phillyreoides*	Fresh	Pericarp	69.531 ± 0.302 ^h^	261.449	266.229 ± 5.132 ^f^
	Freeze-dried		83.110 ± 0.500 ^k^	78.662	396.563 ± 5.774 ^c^
	Fresh	Flower	53.903 ± 0.641 ^c^	184.012	217.281 ± 4.041 ^g^
	Freeze-dried		79.675 ± 0.902 ^i^	83.205	328.563 ± 1.732 ^d^
	Fresh	Leaf	67.287 ± 0.763 ^g^	261.449	289.541 ± 4.509 ^e^
	Freeze-dried		82.177 ± 1.224 ^jk^	157.535	385.229 ± 2.887 ^c^
	Fresh	Peduncle	36.029 ± 0.835 ^a^	261.433	112.665 ± 0.404 ^j^
	Freeze-dried		56.723 ± 0.391 ^d^	145.794	199.254 ± 5.196 ^h^
*Mimosa* sp.	Fresh	Pericarp	61.823 ± 1.180 ^f^	246.149	316.563 ± 1.528 ^d^
	Freeze-dried		85.674 ± 0.426 ^l^	50.504	486.563 ± 6.083 ^a^
	Fresh	Flower	60.080 ± 0.641 ^ef^	142.851	329.229 ± 4.933 ^d^
	Freeze-dried		80.070 ± 0.258 ^ij^	66.998	381.896 ± 0.611 ^c^
	Fresh	Leaf	58.285 ± 0.426 ^de^	154.795	332.563 ± 5.158 ^d^
	Freeze-dried		71.439 ± 1.075 ^h^	106.4	425.229 ± 4.950 ^b^
	Fresh	Peduncle	36.892 ± 0.098 ^a^	148.011	191.483 ± 9.809 ^i^
	Freeze-dried		46.706 ± 0.391 ^b^	143.321	323.896 ± 5.119 ^d^

Mean ± standard deviation is shown. Analysis of variance (ANOVA) and Tukey’s test were performed (*n* = 9, *p* < 0.05); different letters indicate significant differences in columns. ^1^ Response observed with an extract concentration of 100 μg/mL.

**Table 5:** Antioxidant activity of freeze-dried and fresh *P. calyculatus* organs using the ABTS·^+^ technique.

Host	Type of Process	Plant Organ	Inhibition % ^1^	IC_50_ μg/mL	ABTS·^+^ (μM TE/g DW-FW)
*F. phillyreoides*	Fresh	Pericarp	70.977 ± 0.912 ^f^	284.0	418.705 ± 1.528 ^f^
	Freeze-dried		89.765 ± 0.895 ^g^	120.481	523.135 ± 5.44 ^b^
	Fresh	Flower	73.128 ± 0.273 ^f^	241.370	446.049 ± 3.786 ^e^
	Freeze-dried		81.756 ± 0.380 ^i^	142.263	482.866 ± 9.954 ^de^
	Fresh	Leaf	60.984 ± 1.664 ^d^	228.510	241.790 ± 2.649 ^i^
	Freeze-dried		85.961 ± 0.127 ^h^	201.521	515.434 ± 2.517 ^b^
	Fresh	Peduncle	41.718 ± 0.136 ^a^	362.315	128.046 ± 5.508 ^l^
	Freeze-dried		52.528 ± 0.777 ^c^	201.521	206.400 ± 5.69 ^j^
*Mimosa* sp.	Fresh	Pericarp	83.058 ± 0.273 ^gh^	156.102	482.866 ± 1.528 ^d^
	Freeze-dried		94.878 ± 0.246 ^j^	106.313	557.083 ± 1.732 ^a^
	Fresh	Flower	73.420 ± 0.478 ^f^	219.769	332.766 ± 2.050 ^g^
	Freeze-dried		92.717 ± 2.233 ^ij^	131.393	530.207 ± 3.215 ^b^
	Fresh	Leaf	81.875 ± 2.014 ^g^	186.785	452.563 ± 5.132 ^e^
	Freeze-dried		90.523 ± 0.136 ^i^	139.051	500.083 ± 5.292 ^c^
	Fresh	Peduncle	45.169 ± 0.492 ^b^	238.080	141.433 ± 5.859 ^k^
	Freeze-dried		64.728 ± 0.827 ^e^	225.007	307.278 ± 9.124 ^h^

Mean ± standard deviation is shown. Analysis of variance (ANOVA) and Tukey’s test were performed (*n* = 9, *p* < 0.05); different letters indicate significant differences in columns. ^1^ Response observed with an extract concentration of 100 μg/mL.

**Table 6:** Hypoglycemic activity of different organs of *P. calyculatus*.

Host	Type of Process	Plant Organ	α-Glycosidase Inhibition % ^1^	IC_50_ μg/mL
*F. phillyreoides*	Fresh	Pericarp	41.385 ± 0.552 ^e^	120.816
	Freeze-dried		53.908 ± 0.153 ^c^	98.750
	Fresh	Flower	28.952 ± 1.538 ^gh^	172.699
	Freeze-dried		33.689 ± 0.512 ^f^	148.416
	Fresh	Leaf	50.562 ± 0.577 ^d^	98.888
	Freeze-dried		50.600 ± 0.577 ^d^	98.814
	Fresh	Peduncle	41.052 ± 0.577 ^e^	121.796
	Freeze-dried		18.590 ± 1.025 ^i^	268.961
*Mimosa* sp.	Fresh	Pericarp	40.718 ± 0.552 ^e^	122.795
	Freeze-dried		58.459 ± 0.154 ^c^	85.530
	Fresh	Flower	33.689 ± 2.995 ^f^	148.416
	Freeze-dried		59.473 ± 0.857 ^b^	84.071
	Fresh	Leaf	27.768 ± 2.893 ^h^	180.063
	Freeze-dried		41.681 ± 0.577 ^e^	119.958
	Fresh	Peduncle	17.999 ± 1.025 ^i^	277.793
	Freeze-dried		30.728 ± 0.940 ^g^	162.718
(+) Control	Acarbose		80.139 ± 0.391 ^a^	62.047

Mean ± standard deviation is shown. Analysis of variance (ANOVA) and Tukey’s test were performed (*n* = 9, *p* < 0.05); different letters indicate significant differences in columns. ^1^ Response observed with an extract concentration of 100 μg/mL.

## Data Availability

The original contributions presented in this study are included in the article. Further inquiries can be directed to the corresponding author.
